# Myofibroblastic sarcoma in breast: a case report and literature review

**DOI:** 10.3389/fonc.2024.1366546

**Published:** 2024-05-13

**Authors:** Zixi Deng, Chuan Xia, Yuechong Li, Yongchao Luo, Songjie Shen

**Affiliations:** ^1^ Department of Breast Surgery, Peking Union Medical College Hospital, Peking Union Medical College, Chinese Academy of Medical Sciences, Beijing, China; ^2^ Department of Pathology, Peking Union Medical College Hospital, Chinese Academy of Medical Sciences and Peking Union Medical College, Beijing, China

**Keywords:** myofibroblastic sarcoma, myofibrosarcoma, breast, myofibroblast, immunohistochemistry

## Abstract

Myofibroblastic sarcoma is a malignancy in which myofibroblasts are the main component, with a very low incidence. In this study, we report a case of low-grade myofibroblastic sarcoma (LGMS) in the breast. After the diagnosis of LGMS, the patient received a mastectomy. The patient showed no relapse or progression during the follow-up time of 3 months following the operation. LGMS in the breast is extremely rare, and the limited experience with its diagnosis and treatment brings obstacles to doctors. Therefore, this report summarizes the preoperative diagnosis, treatment, and prognosis of breast LGMS through a literature review.

## 1 Introduction

Myofibroblasts are primarily found in reactive granulation tissue, they are generally secondary components of malignancies, reflecting the response of the host stromal tissue to malignant cells. However, malignancies in which myofibroblasts are the main component are rare (1). Low-grade myofibroblastic sarcoma (LGMS) was first reported by Mentzel et al. in a case series of 18 cases in 1998, and it was classified as a distinct type of soft-tissue tumor by the World Health Organization in 2002 (2, 3). LGMS mainly occurs in the head and neck but can also occur in the bones, trunk, limbs, retroperitoneal, and other parts, LGMS in the breast is extremely rare (4–6). About 20 patients with LGMS of the breast have been previously reported in the literature. In this report, we report a rare case of breast LGMS. The patient underwent a right mastectomy, and no recurrence or metastasis has been found so far.

## 2 Case presentation

**Figure 1:**
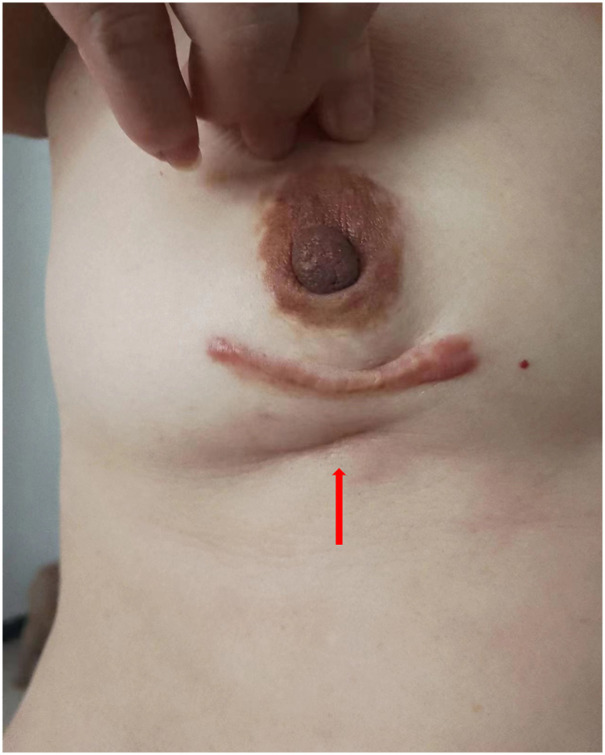
A 1.5cm mass at the 5 o’clock position of the right breast, with the boundary unclear and the local skin sunken (shown by arrow).

**Figure 2:**
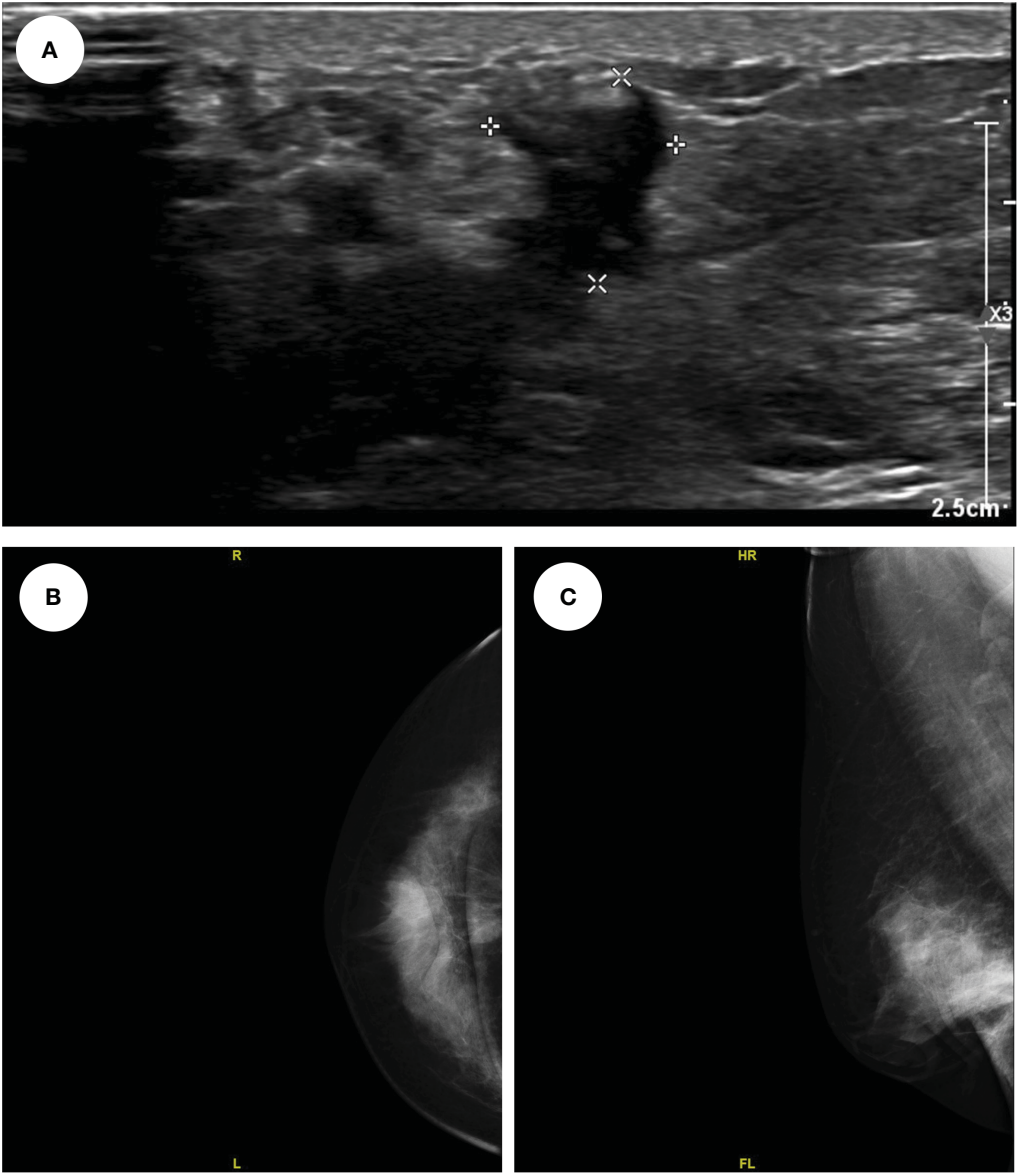
The ultrasound showed a 1.5*0.9*1.0cm hypo echoes irregular mass located in the 5 o’clock position of the right breast **(A)**; The mammogram showed a mass in the inner lower quadrant of the right breast (CC, **B**); (MLO, **C**).

**Figure 3:**
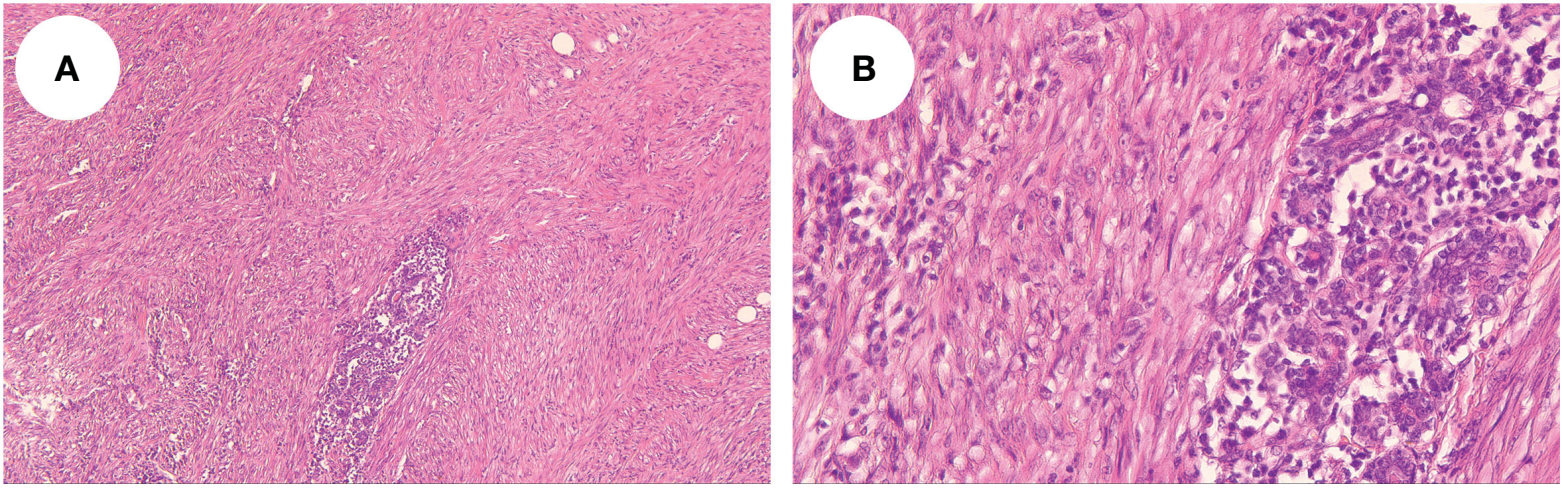
Microscopy showed that the spindle tumor cells were arranged in sweeping fascicles (HE, × 10, **A**); (HE, × 40, **B**).

**Figure 4:**
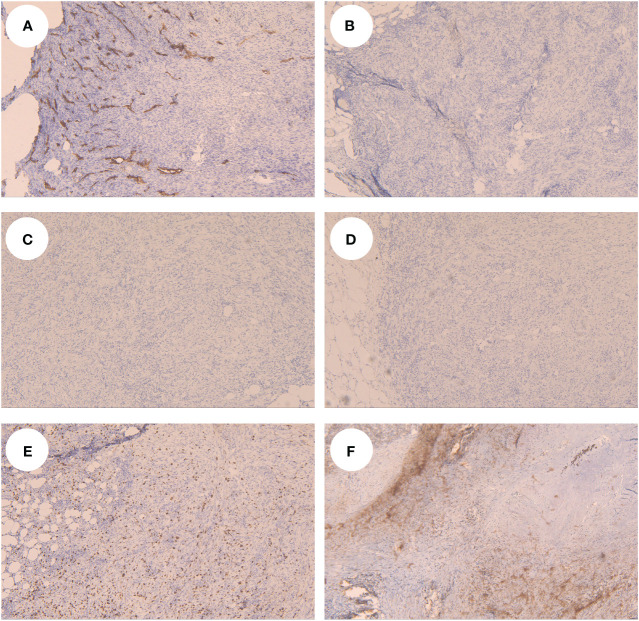
Tumor cells showed CD34 positive in the vessel in IHC **(A)**, showed CK5/6 **(B)**, CK14 **(C)** and Desmin **(D)** negative, Ki-67 **(E)** and SMA **(F)** showed partial positive.

A 54-year-old woman found a palpable, non-tender mass of the right breast, which she first noted on self-examination 9 months earlier. She denied any nipple discharge, itching, skin redness, and swelling of her breast. She underwent a right breast mass excision in July 2019, and the biopsy was fibroadenoma. She had no family history of breast cancer. On physical examination, a 1.5cm nodule was palpable at the 5 o’clock position of the right breast, with the obscured margin and the local skin retraction (**Figure 1**). There was no evidence of lymphadenopathy. Her laboratory tests are normal, and the tumor marker showed no abnormalities. Ultrasound reported a 1.5 ×0.9×1.0cm hypoechoic irregular mass located in the 5 o’clock position of the breast with a surgical scar on the skin. The aspect ratio of the node was greater than 1, and no clear blood flow signal was seen (**Figure 2A**). A mammogram confirmed the presence of a mass in the inner lower quadrant of the right breast (**Figures 2B, C**). Ultrasound-guided core biopsy of the lesion revealed a low-grade malignant spindle cell tumor with necrosis and peripheral lymphocyte infiltration. She underwent a right mastectomy on July 06, 2023. The patient’s postoperative recovery was uneventful, and she was discharged home after three days. Examination of the resected specimens revealed 1.8cm gray and white nodules with unclear boundaries. Under hematoxylin-eosin (HE) staining, spindle tumor cells can be seen as arranged in sweeping fascicles (**Figures 3A, B**). Combined with immunohistochemistry, LGMS was diagnosed, and the surgical margins were negative. Immunohistochemical (IHC) staining showed (**Figure 4**): CD34 (Vascular+) (**Figure 4A**), CK5/6 (-) (**Figure 4B**), CK14 (-) (**Figure 4C**), Desmin (-) (**Figure 4D**), Ki-67 (index 30%) (**Figure 4E**), SMA (partial+) (**Figure 4F**), AE1/AE3 (-), CD31 (Vascular+), ALK-D5F3 (-), ALK-D5F3 (NC) (-), ALK-D5F3 (PC) (+), β-catenin (-), S-100 (-), STAT6 (-), P63 (scattered +), EGFR (-), GFAP (-). Following mastectomy, the patient received no chemotherapy or radiation therapy. Furthermore, it is noteworthy that the patient maintained a positive attitude and collaborative spirit throughout the entire treatment process. Due to concerns about recurrence, the patient strongly requested a mastectomy, demonstrating resilience and optimism despite the challenging nature of the procedure, which played a pivotal role in the successful management of her condition. Additionally, we utilized physical examinations and imaging studies (ultrasound and mammography) during subsequent follow-up visits to ensure treatment continuity. She exhibits no evidence of metastasis or local recurrence after 3 months of follow-up.

## 3 Discussion

We reviewed previous case reports of breast LGMS, and **Table 1** shows the relevant studies we reviewed, in which one case focused on the imaging features of breast LGMS (case 15), and the rest of the cases described the histological features, treatment and prognosis. LGMS consists of fibrosarcoma-like spindle tumor cells arranged in sweeping fascicles, sheets, or storiform whorls (21). Tapered or ovoid nuclei with deep staining have small nucleoli surrounding homogenous chromatin and display light to moderate atypia with slightly increased proliferative activity. The tumor cells have small or moderate amounts of eosinophilic cytoplasm with distinct cell boundaries. The peripheral stroma is collagenous and/or has a myxoid background, and necrosis occurs in a minority of cases. The mitotic activity ranged from 8 to 35 per 10 high power field (HPF), but abnormal mitotic figures are rare (22). A superficial biopsy may have a strong resemblance to a myofibroma. However, the histological features of invasive growth of tumor cells are different from those of myofibromatosis and similar diseases such as nodular fasciitis or inflammatory myofibroblastic tumor (IMT) and more similar to the growth pattern of fibromatosis (23).

**Table 1:** Cases of myofibroblastic sarcomas of the breast.

Case	Background	Age/Sex	Medical history	Size (cm)	Imaging	Operation	RT/CT	IHC	Following up (months)
1/(7)	Nm	55/F	1W	2	**Ultrasound**: the neoplasm is found to consist of confluent nodules forming an overall mass	Mastectomy, lymphadenectomy	CT	Vimentin (+), SMA (+), others (–)	Recurrence (1); Pleuropulmonary metastasis and dead (11)
2/(8)	Nm	59/F	Nm	2.3	Nm	Mastectomy, lymphadenectomy	RT	Vimentin (+), SMA (+), S100 (+), CD68 (+), others (-)	Alive (20)
3/(9)	Nm	60/M	1M	2.5	Nm	Mastectomy, lymphadenectomy	No	Vimentin (+), SMA (+), others (-)	Recurrence and alive (120)
4/(5)	Nm	72/F	Nm	3.4	Nm	Local excision	No	SMA (+), MSA (+), desmin (+), others (-)	Recurrence (5); Lung metastasis (12)
5/(22)	Nm	51/F	3M	22	**Ultrasound**: a huge tumor replacing almost the entire breast tissue	Mastectomy	RT and CT	Vimentin (+), SMA (+), others (-)	Alive (24)
6/(1)	Nm	46/M	6M	2.2	**Mammogram**: a lobular mass with partially circumscribed edges in the upper inner quadrant, measuring 22*21*16 mm	Mastectomy	No	Vimentin (+), SMA (+), fibronectin (+), others (-)	Alive (12)
7/(10)	Nm	46/F	Nm	4	Nm	Local excision	Nm	Vimentin (+), SMA (+), CD34 (+), others (-)	Recurrence (12)
8/(11)	Nm	81/F	2W	4.2	**Mammogram**: the lesion is identified as a confluent mass lesion measuring 4.2 *3.5*2.5 cm	Mastectomy	RT	Vimentin (+), SMA (+), Bcl-2 (+), others (-)	Pleuropulmonary metastasis and alive (14)
9/(12)	Nm	37/F	Nm	2	**Ultrasound**: solid echo mass. **MRI**: ring enhancement pattern, signal intensity/time curve: fast initial phase, plateau delayed phase	Mastectomy	No	Vimentin (+), SMA (+), CD34 (+), ALK (+), Ki67 30%, others (-)	Alive (3)
10/(13)	Nm	61/F	1Y	6	**Mammogram**: a lobular mass with high density, obscured margin	Nm	Nm	Vimentin (+), Actin (+), CD34 (+), ALK (+), others (-)	Bone metastasis (Nm)
11/(14)	Nm	57/F	Nm	Nm	Nm	Nm	Nm	SMA (+), others (-)	Pulmonary and brain metastasis, dead (12)
12/(14)	Nm	82/F	Nm	Nm	Nm	Nm	Nm	SMA (+), desmin (+), calponin (+), others (-)	Dead (15)
13/(15)	Arising in fibroadenoma	61/F	8M	5	**Mammogram**: a 3.5-cm-sized well-defined nodular mass with high density at 11 o’clock position of center breast	Local excision	No	Vimentin (+), SMA (+), fibronectin (+), calponin (+), others (-)	Alive (24)
14/(16)	Nm	47/F	6M	3.8	**Ultrasound**: 3.8*2.1cm hypoechoic mass at 3 o’clock position of center breast	Local excision	No	Vimentin (+), SMA (+), CD34 (+), Ki67 30%, others (-)	Alive (3)
15/(6)	Nm	Nm/F	1Y	6.2	**Mammogram**: a lobulated mass, high density, multiple angular and poorly circumscribed margins, a lack of encapsulation	Nm	No	Nm	Bone metastasis (11)
16/(17)	RT associated	49/F	1M	2.8	**Mammogram**: microcalcifications in the upper outer quadrant of the center breast and the upper inner quadrant of the right breast. **Ultrasound**: a hypogenic area of poorly defined limits and several enlarged axillary lymph nodes	Local excision	No	SMA (+), MSA (+), fibronectin (+), CD99 (+), calponin (+), others (-)	Alive (2)
17/(18)	Mastectomy and ALND with CT	53/F	2M	Nm	Nm	No	No	Nm	Lung metastasis (2)
18/(30)	Nm	62/F	Nm	2	**Mammogram**: 2-cm-sized well-defined nodular mass with high density	Local excision	No	Vimentin (+), SMA (+), Ki67 20%, others (-)	Recurrence (Nm)
19/(19)	BCS with RT and CT	49/F	Nm	3	**Ultrasound**: an oval heterogeneous mass measuring 30 mm in the right retropectoral area	Mastectomy	RT	Vimentin (+), SMA (+), Ki67 43.1%, others (-)	Nm
20/(20)	Nm	56/F	Nm	3	**Ultrasound**: 3*2cm hypoechoic mass at 7 o’clock position of right breast	Local excision	Nm	Vimentin (+), SMA (+), CD34 (+), Ki67 10%, others (-)	Nm
21/ (present case)	Arising in fibroadenoma	54/F	9M	1.5	**Ultrasound**: a 1.5 *0.9*1.0cm hypoechoic irregular mass located in the 5 o’clock position of the breast with a surgical scar on the skin. **Mammogram**: a mass in the inner lower quadrant of the right breast	Mastectomy	No	CD34 (+), SMA (+), Ki67 30%, CD31 (+), ALK-D5F3 (+), P63 (+), others (-)	Alive (3)

Nm, no mentioned; RT, radiotherapy; CT, chemotherapy; IHC, immunohistochemistry; ALND, axillary lymph nodes dissection; BCS, breast conservation surgery; F, female; M, male; Y, year; M, month; W, week.

Widely accepted for diagnosing LGMS, an electron microscope is required to demonstrate specialized ultrastructural organelles, including abundant rough endoplasmic reticulum, fibronexus junctions, and fibronectin fibrils (5). Among them, fibronexus was not found in smooth muscle cells and fibroblasts. It has been regarded as the most specific ultrastructural marker for myofibroblasts, connecting the myofibroblast to the extracellular matrix (1). The cell-to-matrix junction consists of myofilament and fibronectin filament systems converging on a discrete cell-surface plaque. Calponin and caldesmon, the main components of smooth muscle filaments, are regulators of smooth muscle contraction, and they can interact with actin to inhibit the ATPase activity of muscle actomyosin, thereby regulating muscle contraction (24). Some studies have also found that IMT and LGMS cells, following immunohistochemical staining, suggest diffusely positive for actin-associated proteins and fibronectin (25). These proteins were noted to be mainly distributed in the cytoplasm of tumor cells, with a tendency to extend into the extracellular space. Actin-associated proteins generally include α-smooth muscle actin (α-SMA), muscle-specific actin (MSA), and calponin, etc. As mentioned above, actin exists in myofilaments to regulate the contractile function of muscles. Proteins that are stained positive may be able to prove that myofibroblasts contain myofilaments. Fibronectin staining of spindle cells with extracellular extension might be equivalent to fibronectin fibrils known at the ultrastructure of myofibroblasts (25). Therefore, the combined expression of these markers may immunohistochemically reflect the characteristic cell-to-matrix ultrastructural components of myofibroblasts.

In immunohistochemical studies of LGMS, α-SMA and vimentin can be positive in most cases, but desmin can be both positive and negative (21). In this case, α-SMA staining showed positive, vimentin and desmin staining showed negative. Although SMA is regarded as the most specific and frequently expressed marker of myofibroblasts, a small percentage of cases express only SMA or desmin. In addition, calponin can often be positive, and CK/CD34/S-100 is often negative, as in this case. Focal expression of S-100 protein and CD34 has been observed in a minority of cases (23). Furthermore, H-caldesmon is usually expressed positively in leiomyosarcoma but negatively in LGMS, which plays a vital role in the differential diagnosis of the two. Moreover, CK expression is commonly seen in other myofibroblastic proliferations, like the inflammatory myofibroblastic tumor (4).

The mammograms of breast LGMS are generally irregular shapes and high density, with a lack of encapsulation (6). LGMS of the breast are tumors of stromal origin and are etiologically more likely to have hematologic metastases than to have lymphoid metastases compared to epithelial original breast cancers. Therefore, mammograms of advanced-stage breast cancers usually find interstitial edema and opacity of the subcutaneous fat layer (6). On the other hand, there are no signs of interstitial edema and swollen axillary lymph nodes in LGMS. We reviewed cases of breast LGMS, and a few patients underwent computed tomography (CT) and magnetic resonance imaging (MRI), so they could not provide sufficient imaging evidence for diagnosis.

Breast LGMS seems to have a higher incidence of distant metastasis. In the cases we reviewed, almost all patients underwent surgical treatment, with 31.3% of postoperative recurrences and 18.8% of distant metastases. In terms of LGMS at other sites, previous studies have calculated a local recurrence rate of 13.3% to 44.4% (5, 26, 27). In the previous study by Munehisa Kito, 24 patients with LGMS were counted and analyzed statistically (4). Among them, 22 patients underwent surgical treatment, the results showed that the local recurrence rate was 20%, and the incidence of distant metastasis was 4.2%. In a population-based study on LGMS, 49 LGMS patients in the Surveillance, Epidemiology, and End Results Program (SEER) database had survival data with 3-year and 5-year overall survival (OS) of 75.0% and 71.6%, and disease-specific survival (DSS) of 80.0% and 76.3%, respectively. Of these, 93.9% underwent surgery (28). Additionally, a univariate analysis of prognostic outcomes was performed, and the results showed that only tumors larger than 5cm in diameter had a higher local recurrence rate (29). However, this conclusion may not apply to breast LGMS, as shown in **Table 1**, patients with local recurrence had tumors smaller than 5 cm. Tumor size is not one of the critical factors affecting the long-term prognosis of breast LGMS patients. Therefore, for the prognostic analysis of breast LGMS, more cases and data analysis are still needed.

As for the treatment of LGMS, the benefits of radiotherapy and chemotherapy for patients after surgery are unclear (28). Almost all patients have undergone surgery, and no patients have received radiotherapy or chemotherapy alone. Thus, the therapeutic effect of radiotherapy or chemotherapy cannot be evaluated separately (22, 26, 30). In Y. Xu’s study, 96 patients with LGMS were included in the SEER database, of whom 89.6% had received surgery, 29.2% had received radiotherapy, and 10.4% had received chemotherapy (31). According to the results, chemotherapy and radiotherapy had no significant benefit on patients’ OS and DSS. Only For unresectable cases, radiotherapy may be a treatment option (4). However, there have been reports of cases of recurrence outside the irradiated area after radiotherapy in LGMS patients, likely related to the pattern of invasive tumor growth. Thus, the choice of radiotherapy may be considered in some cases. In summary, surgery may currently be the primary treatment for LGMS patients, while the prognosis and benefits of radiotherapy and chemotherapy are still unknown. This study comes with its limitations, a three-month follow-up period may not be sufficient to fully ascertain the long-term prognosis of LGMS. Future studies with extended follow-up periods are imperative to gain a more comprehensive understanding of the recurrence patterns and overall prognosis for patients with LGMS.

## 4 Conclusions

The diagnosis of LGMS still relies on discovering special ultrastructure under electron microscopy, and the specificity of immunohistochemistry and imaging methods is still insufficient. According to the current literature, surgery is the most important treatment. Notably, LGMS occurring in the breast is a tumor with a high rate of recurrence and a higher incidence of distant metastasis relative to other sites. The currently summarized data on the clinical manifestations, treatment, and prognosis of LGMS mainly come from other sites, and the data of LGMS in the breast need to be further accumulated.

## Data availability statement

The original contributions presented in the study are included in the article/supplementary material. Further inquiries can be directed to the corresponding author.

## Ethics statement

Ethical approval was not required for the study involving humans in accordance with the local legislation and institutional requirements. Written informed consent to participate in this study was not required from the participants or the participants’ legal guardians/next of kin in accordance with the national legislation and the institutional requirements. Written informed consent was obtained from the individual(s), and minor(s)’ legal guardian/next of kin, for the publication of any potentially identifiable images or data included in this article.

## Author contributions

ZD: Writing – original draft, Methodology, Conceptualization. CX: Writing – original draft, Resources. YLi: Writing – review & editing. YLu: Writing – review & editing. SS: Writing – review & editing, Project administration, Methodology, Conceptualization.
